# Psychiatry

**Published:** 1905-01-21

**Authors:** 


### Progress in Medicine and Surgery.

### PSYCHIATRY.

(*Continued from page* 285.)

**Arrest of the Progress of Insanity, with Permanent Partial Dementia.**—There are occasions when a broad distinction can be justifiably made between psychoses which lead to complete dementia and those which suffer arrest, leaving the patient in a state of mild and partial dementia permanently. Professor Dana, of Cornell University, records such a case [6] which is worthy of note. The patient's symptoms were elaborately studied and tested at three different periods, by laboratory methods and instruments. The family history showed a neuropathic element. The mother of the patient developed mania at the age of 40 years, followed by a "kind of neurasthenic insanity characterised by mysophobia and refusal of food." His father was a man of good health. At the age of 19 the patient suffered from mental depression, and was sent to Europe, whence he returned recovered in about six months. At the age of 23 he again had an attack of depression, and wounded himself, accidentally it was thought, with a pistol. For six months he refused food and preserved a stubborn silence. Then he began to manifest delusions of an active character, which, however, passed off in the course of the next two years, so that he was then, at the age of 26 years, able to resume his proper place in family and social life. He remained well for 12 years after, the only indications of disorder being the appearance, at times, of a few delusions of a prominent character. His mood was eccentric and suspicious, his habits were extravagant, and he was looked upon as a person fond of books and mechanical inventions. At the age of 38, after having been perfectly immune from attacks of mental disorder for 12 years, he suddenly developed an attack of acute maniacal excitement, with violence and delusions of grandeur, so that he had to be placed in an asylum. Here he passed through a stage of stupor, after which partial recovery followed. In two months he attained to a state of recovery, with arrest of symptoms, but this stage of arrest was marked by a slight and just perceptible dementia. Thus he could go about the asylum and take reasonably good care of himself. His sense of locality and of his surroundings was vague and imperfect; he could not recognise the nature of the institution where he dwelt, the physicians who attended him, or the relatives who visited him. His ideas were grandiose; he called himself Vanderbilt, the Sultan of Turkey, and Mr. X. Domestic sentiments and ties seemed to have vanished, while his egotism was intense. He busied himself in calculations with figures, and designed diagrams illustrating ridiculous and even impossible theories. He did not care for or take any interest in the current events of the day, and did not look at the newspapers or illustrated journals except as suggesting to his mind something pertinent to his own calculations. He could play billiards and draughts fairly well if his attention was occasionally

"coached." He was most cleanly in person and in toilet. "His manner was that of an active man, much absorbed in literary, inventive, and mathematical work. For six years he continued thus." It became important to know whether he was imperceptibly passing into dementia or equally imperceptibly recovering. Careful tests with physiological apparatus were made to ascertain the exact conditions of motor function, special and general senses, reaction time, and the elementary and more complex psychical functions. These examinations were conducted in March 1899, July 1899, and May 1902. The higher senses were normal, but the mental processes were found to be slightly incoherent. The intervals between the successive examinations and the time that has elapsed since then did not develop the slightest change of symptoms or character. The process of disease in the brain, concludes Professor Dana, having "sloughed off" certain psychical functions, ceased to progress further; and a permanent state of partial dementia was left. Perhaps in time further degeneration might occur as natural senile decay came on. The disorder in the brain and the permanent destruction of brain tissue involved belonged in all probability to one or more of Flechsig's "association areas" of the brain.

**Frontal Lobe Tumours and Insanity.**—While the above paragraph deals with a destructive lesion of the highest or association-centres of the brain, the following refers to the mental disorder and dementia resulting from the growth of tumours in the frontal lobe of the brain. The classical researches of Von Brüns, Bianchi, Höniger, and others have shown that frontal lobe tumours produce effects on the psychical functions varying with their site and the extent of their involvement of the frontal cortex. Auerbach [7] reports a specially interesting case. The patient was a woman 48 years of age who had always suffered from migraine. Usually she had been industrious, but she began to neglect her work, became irritable, complained of fatigue, had increasing headache, and often refused to leave her bed. From time to time she vomited. She lost interest in her surroundings, grew apathetic, refused to eat, slept considerably, and was wet and dirty in her habits. The knee jerks were increased but there was neither ankle clonus nor Babinski's phenomenon. Intelligence seemed to be considerably obscured and memory for recent events was much impaired. There was no tendency to jokes or oddities of speech. Tremor of the hands was frequent. She suffered once from an apoplectiform attack, during which she was unconscious for half an hour; and on recovering it was noticed that the pupils were dilated, there was difficulty in fixing the eyes, and in swallowing. She complained of pain in the spinal column. Ophthalmoscopic examination of the eyes showed choked discs. Later she developed stiffness and pain in the neck, grew comatose, and died. The autopsy revealed the following conditions: the frontal lobes of the brain were found flattened, the dura mater was adherent to the brain in this region, and the local veins were markedly congested, especially over the left frontal lobe. The base of both frontal lobes was occupied by a tumour about the size of an apple, extending from the anterior perforated substance forwards, nodular, of the consistence of liver, and of a pink to dark red colour. It proved to be a fibro-sarcoma, probably arising from the basal dura mater. At first Professor Edinger, who saw the case, thought of precocious senile dementia, or arterio-sclerotic brain disease. Later, tumour of the frontal lobe was suspected, but the only symptoms were the headache and the psychical disturbances. Of the latter, the impairment of recent memory, the loss of power of attention, apathy and loss of all initiative in voluntary action, were the more prominent features. Auerbach believes that in the human brain the frontal association centres are to be regarded as the most important centres of association. Among the interesting symptoms are the apoplectic form of attack, the occurrence of choked discs, the absence of disturbance of equilibrium, and the absence of the tendency to make jokes. The tremor of the hand is a peculiar and striking feature, rare in cases of this sort. Among the negative symptoms, temperature was normal and smell undisturbed. Operation could not be performed, as the relatives refused consent. The association of mental symptoms constituting dementia formed the central feature in this case.

**Insanity in the Malay and Egyptian Races.**—Professor Kraepelin, of Heidelberg, has availed himself of an opportunity to study insanity among the Malay native patients at the Buitenzorg Asylum in Java. Patients of European birth in Java, says Kraepelin, show the same clinical pictures as at home, the exceptional rarity of senile dementia being, as in other colonies, due to the character of the population. The natives use no intoxicating liquor, and so alcoholic insanities do not occur. Insanity due to opium does not occur in Java, nor are they seen (according to Dr. Ellis) in Singapore, where the population is Chinese, and opium-eating is widespread. Malarial psychoses were not observed among the patients. General paralysis of the insane and brain-syphilis were not found in a single one of 370 insane natives, though there were eight such cases among 50 European insane—a difference which accords with experience in other lands. This is interesting in accordance with the facts about alcohol and the great prominence given to syphilis in the etiology of general paralysis by Krafft-Ebing and his followers. Dementia præcox was exceedingly frequent among the natives; manic depressive insanity was rare. States which can with fair accuracy be called psychical epilepsy were frequent. "The symptoms of insanity in the natives were not clean-cut; katatonic signs, hallucinations of hearing, and systematised delusions were almost absent. Prodromal depression was slight, and terminal dementia consisted in moderate mental confusion with dulness." Transitory frenzies were common. The often described *amok* and *latah*, according to Kraepelin, are peculiar forms of the known insanities. *Latah* is an imitative automatism with coprolalia, arising from sudden emotional excitement, with complete preservation of consciousness, reminding us of hysteria. *Amok*, on the other hand, is not a single affection, but includes impulsive violent acts with impaired consciousness. Some of these are homicidal and suicidal acts (of early katatonia), most are the outcome of psychical epilepsy, and are connected with other signs of the epileptic dreamy state. A few cases, however, cannot be thus accounted for, and the possibility of larvated attacks of malaria must be considered. Kraepelin's general conclusion is that the natives of Java show none but recognised forms of insanity, modified in development of symptoms even as the people's mental development is at a lower stage. Dr. John Warnock in his annual report on the Egyptian Government Hospital for the insane[9] draws attention to the character of lunacy among the Egyptian native population. The characteristic insanity of the people is that due to *haschish*, a vegetable drug, allied to Indian hemp, which the natives consume in large quantities; and although the importation of it is forbidden by the Egyptian Government large quantities of it find their way into Egypt. Of 495 patients admitted to the Cairo Asylum during the year, 74 were "hasheesh insanities," in the form of maniacal excitement, delirium and intoxication, melancholic depression, weakmindedness, and chronic dementia. Pellagious insanity was responsible for 52 cases, and general paralysis of the insane for 35. The proportion of haschish patients yearly is steadily decreasing, and this is attributed to the price of the drug having steadily risen. Thus the percentage of cases was $32\frac{2}{3}$ in 1897, 25 in 1898, $23\frac{1}{2}$ in 1899, 25 in 1900, 21 in 1901, and 22 in 1902, while it had fallen to 18 per cent. in 1903.

[6] Cornell Univ. Studies, Dept. of Neurol., 1904. [7] Deuts. Zeitch. für Nervenheilk., Bd. 22, H. 4. [8] Centralb. für Neur. und Psychiatrie, July, 1904. [9] Report of the Egyptian Government Hospital for the Insane, 1904.

